# Oligosaccharide-based quality evaluation of Atractylodis rhizome and a strategy for simplifying its quality control

**DOI:** 10.1186/s13065-019-0605-8

**Published:** 2019-07-12

**Authors:** Dan Zhuang, Jing Qin, Hui-yang Wang, Yi Zhang, Chun-yao Liu, Qing-qing Ding, Guang-ping Lv

**Affiliations:** 1 School of Pharmaceutical Sciences, Nanjing Tech University, Nanjing, 211816 People’s Republic of China; 2 School of Biotechnology and Pharmaceutical Engineering, Nanjing Tech University, Nanjing, 211816 People’s Republic of China; 3 Department of Geriatric Oncology, Jiangsu Province Hospital, The First Affiliated Hospital with Nanjing Medical University, Nanjing, Jiangsu 210029 People’s Republic of China; 4 National Resource Center for Chinese Materia Medica, Academy of Chinese Medical Sciences, Beijing, 100700 People’s Republic of China

**Keywords:** Atractylodis rhizoma, Fructooligosaccharides, Quantitative method, Chemometrics analysis, Quality control

## Abstract

**Background:**

Atractylodis rhizoma, is the dried rhizomes of *Atractylodes lancea* (Thunb.) DC. or *A. chinensis* (DC.) Koidz. Both of two are pharmacologically and economically important, while with differences in efficacy. Therefore, an authentication system is vital for evaluation the quality and discrimination adulteration of Atractylodis rhizoma. Fructooligosaccharides (FOS), which are regarded as functional ingredients in Atractylodis rhizoma, have not been used for quality control of Atractylodis rhizoma for shortage of reference compounds.

**Results:**

A HPLC-ELSD method was developed for the quantification of FOS in Atractylodis rhizoma. And chemometrics analysis showed that 2 markers including content of degree of polymerization (DP) 12 and total content of DP 3-15 could be used as the main distinctive elements for quality evaluation of Atractylodis rhizome. Actually, the separation and purification of high DP FOS, such as DP 12, is still a challenge because of high polarity. Then DP 5-based qualification evaluation was investigated for quality control of Atractylodis rhizoma. The results showed that *A. lancea* and *A. chinensis* could be clearly separated.

**Conclusions:**

DP 5-based quantification method was credible and effectively adopted for solving the shortage of reference compounds and improving the quality control of Atractylodis rhizoma

## Introduction

Herbal medicine has gained popularity in many countries throughout the years. With the increased trend of usage worldwide, the assessment of safety, quality, and efficacy of these medicines has been an important concern for the public [1]. Atractylodis rhizome is a traditional perennial herb, which has been known and used as medication for a very long time. It is widely distributed in eastern Asia [2]. Korean and Japanese pharmacopoeias have recorded this herb as traditional diuretic and gastric prescriptions [3, 4]. Atractylodis rhizoma origin from dried rhizomes of *A. lancea* (Thunb.) DC. and *A. Chinensis* (DC.) Koidz. documented in Chinese Pharmacopoeia 2015 [5]. It is mainly distributed in Heilongjiang, Hebei, Jiangsu, Zhejiang, Anhui and Hubei province. It has been firstly reported in Shen Nong Ben Cao Jing, the earliest masterpiece about Chinese medicine [6], reputed to invigorate the spleen and eliminate dampness. And it is commonly used for the treatment of rheumatic diseases, influenza, digestive disorders and other diseases in clinical [7]. The multiple components contained in the Atractylodis rhizome contributed to its beneficial effects including a series of sesquiterpenoids, oligosaccharides, polysaccharides, monoterpenes, polyacetylenes, phenolic acids, and steroids [7]. *A. lancea* produced in the Maoshan area of Jiangsu Province is the geo-authentic herb, which is famous for the higher oxygenous sesquiterpenoid content and diversity. However, for ecological destruction and overexploitation, the geo-authentic herb has been endangered in the last few years [8]. Therefor many cultivated varieties were also used as Atractylodis rhizome in the market. There have been studies showed that there are obvious differences of volatile components and oxygenous sesquiterpenoid in *A. lancea* and cultivated varieties [3, 6].

Carbohydrates is one of important medicinal compounds of Atractylodis rhizoma, which possesses various and high biological activities, for instance, enhancing immunity, releasing stress, regulating the enzyme activity, promoting probiotics growth and relieving liver injury etc. [9]. In our previous study, a series of oligosaccharides DP 3-15 were separated from *A. lancea* and identified as FOS, which consisting of linear β-(2,1)-linked fructofuranosyl units mostly carry a terminal single α-(1–2)-linked α-glucopyranosyl unit [10–12]. It is a well-recognized class of prebiotics [13–15], which are dietary ingredients that cannot be digested by human-produced digestive enzymes, yet they provide a health benefit to the host mediated by selectively stimulating the growth and/or activity of one or a limited number of host gut microbiota [16]. It is becoming increasingly clear that FOS can reduce or prevent gastroenteritis, inflammatory bowel disease (IBS), reduce the risk of colon cancer and reduce potentially pathogenic gastro-intestinal bacteria [17]. These activities are in line with the quality of Atractylodis rhizome for both natural and cultured products. Therefore, the level of FOS is an essential marker for quality evaluation of Atractylodis rhizome.

However, the reference compounds of FOS, which are the standards for qualitative and quantitative analysis, are still difficult to separate and purify for high polarity. Up to date, only FOS with DP 3-5 purified by some laboratories have been commercialized [18, 19]. In addition, the chromatographic analysis of FOS is also challenging, since (i) the significantly different solubility, (ii) the absence of high DP standards (DP > 5), and (iii) the absence of chromophores required for UV/Vis detectors. Evaporative light scattering detector (ELSD) is a universal detector, which has been widely applied in detecting analytes without UV absorption. Moreover, ELSD is more sensitive due to gradient elution is available for this detector.

In this study, a HILIC–HPLC method couple with evaporative light-scattering detector (ELSD) was developed for the qualitative and quantitative analysis of 13 FOS in 21 samples including 11 batches of *A. lancea* and 10 batches of *A. chinensis*. Based on the quantification information, chemometrics analysis including principal component analysis (PCA), factor analysis (FA) and hierarchical clustering analysis (HCA) were also applied to discriminate different kinds of Atractylodis rhizome and found out the distinctive components to simplify the quality evaluation procedure. Also, the DP 5-based quantification results were compared with the individual quantification results to evaluate the feasibility and accuracy and provide an alternative quantification method for the quality control of oligosaccharides.

## Materials and methods

### Chemicals and materials

The materials of *A. lancea* and *A. chinensis* were collected from different cultivation locations in China, which are listed in Table 1. Species identification was performed by Associate Prof. Guang-ping Lv. Voucher specimens of the samples were deposited at the School of Pharmaceutical Sciences, Nanjing Tech University, Nanjing, China. The herbs were dried below 50 °C and then ground into fine powder before use.

**Table 1:** Tested samples of *A. lancea* and *A. chinensis*

Producing area	Codes	Producing area	Codes
*A. lancea* (Thunb.) DC., family Compositae		*A. chinensis* (DC.) Koidz., family Compositae	
Jiangsu	A1	Hebei	B1
Jiangsu	A2	Hebei	B2
Jiangsu	A3	Hebei	B3
Jiangsu	A4	Hebei	B4
Jiangsu	A5	Inner Mongolia	B5
Anhui	A6	Inner Mongolia	B6
Anhui	A7	Inner Mongolia	B7
Hubei	A8	Liaoning	B8
Hubei	A9	Liaoning	B9
Henan	A10	Beijing	B10
Yunnan	A11		

**Fig. 1:**
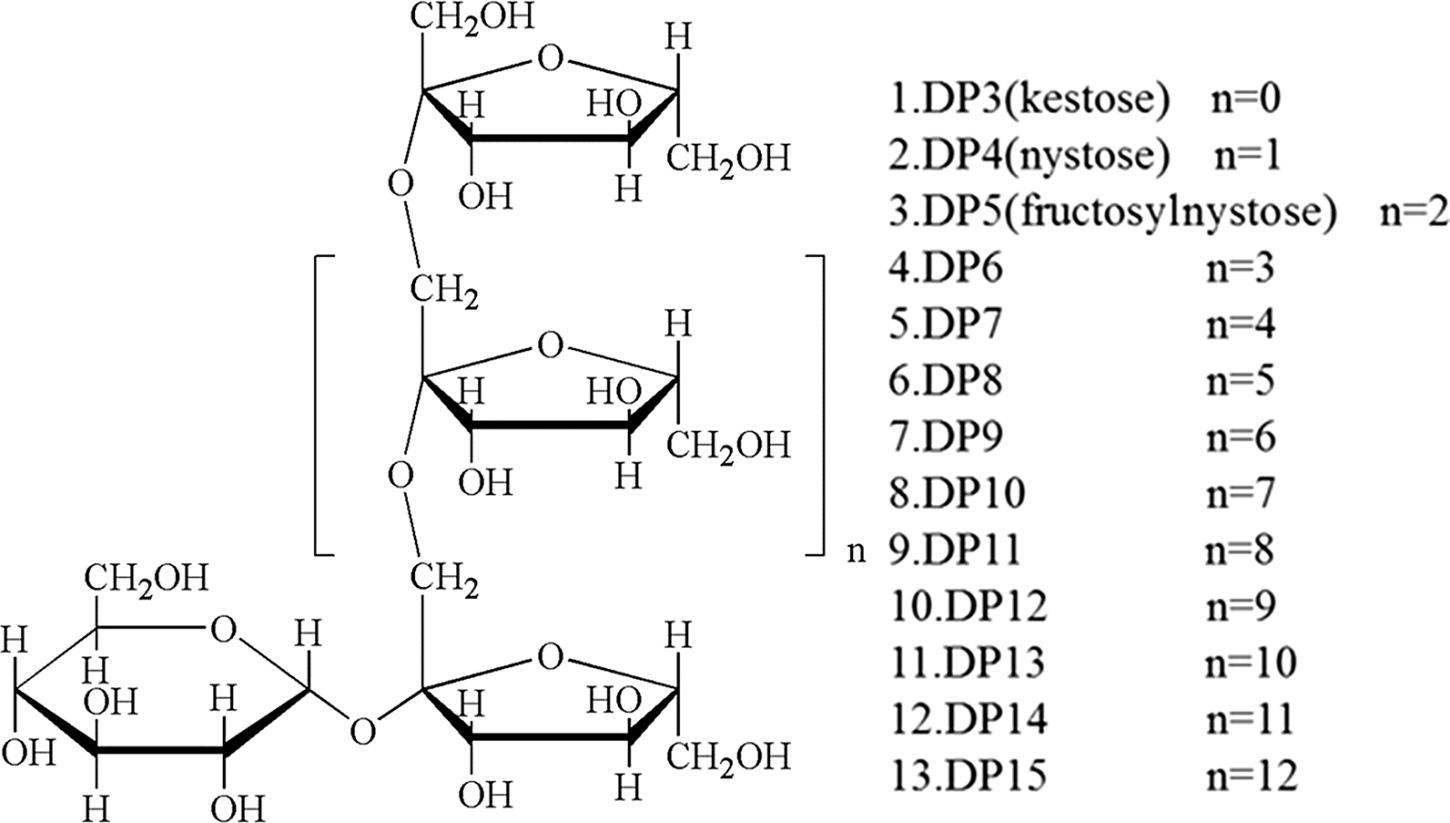
Structures of inulin-type fructooligosaccharides (FOS)

Inulin-type FOS with DP 3-15 (all purities determined by HPLC- ELSD were more than 90%) were separated and purified in our laboratory (Fig. 1). The structures were confirmed by comparing their methylation analysis, MS, and NMR data with the literature [20–22]. Acetonitrile for HPLC was purchased from Merck (Darmstadt, Germany). Deionized water for HPLC was prepared by an Aquaplore 2S system (Aquapro, USA). All other chemicals and reagents were of analytical grade.

### Preparation of standard solutions

Mixed standard stock solution containing inulin-type FOS (DP 3-15) was prepared in 60% ethanol. The concentrations of DP 3-15 were about 3 mg/mL. The standard stock solution was stored in a refrigerator at 4 °C before use. Working standard solutions were prepared from the stock solution by dilution with the appropriate volume of 60% ethanol.

### Sample preparation

Atractylodis rhizoma powders (1 g), passed through the 40-mesh sieve, was extracted by refluxing under the optimized conditions. In brief, the extraction was operated with 20 mL extract solvent of ethanol–water (60:40, v/v) for 2.5 h at 80 °C. All extractions were performed with magnetic stirring. After extraction, appropriate volume of 60% ethanol was added to the extract in order to complement the weight. Then, the extracted slurry was centrifuged at 3500 r/min for 20 min. And 1 mL of supernatant was filtered through a 0.45 μm filter before injection into the HPLC system for analysis.

### HPLC–ELSD analysis

Chromatographic analyses were performed on a Thermo UltiMate 3000 liquid chromatography system (Thermo, USA), equipped with a quaternary pump, an on-line degasser, an auto-sampler, a column temperature controller and UV detectors, coupled with an Alltech ELSD 6000 (Alltech, Deerfield, IL, USA). Data processing was carried out with Dionex™ Chromeleon™ 7.0 software. A gas generation system from Hui Chi Science & Technology Co. (WSC10LP, Shanghai, CN) was applied to provide the nebulizer gas for the ELSD. The drift tube temperature of the ELSD was set at 92.5 °C with the gas flow rate of 2.5 L/min and the value of the gain was 1. The standards and samples were separated with a Waters XBridge Amide column (4.6 × 250 mm id, 3.5 μm) at 30 °C with flow rate of 1.0 mL/min. The mobile phase consisted of water (A) and acetonitrile (B). The gradient was set: 75–68% B at 0–6 min, 68–50% B at 6–21 min, 50–75% B at 21–25 min, 75–75% B at 25–30 min. The injection volume was 5 μL.

### Calibration curves, LOD and LOQ

Standard stock solutions containing reference compounds (DP 3-15) were prepared and diluted to appropriate concentrations for the establishment of calibration curves. At least six concentrations of 13 analyte solutions were injected in triplicate, and then the calibration curves were constructed and their linear ranges were determined. Since ELSD response was nonlinear and the calibration of ELSD could be constructed by a double logarithmic plot as in previous reports, the calibration curves were established by plotting the logarithm peak area versus the logarithm concentration (mg/mL) of each analyte detected by ELSD.

The stock solutions containing reference compounds were diluted with 60% ethanol to appropriate concentrations, aliquots of the diluted solutions were injected into HPLC for determining the limit of detection (LOD) and limit of quantification (LOQ). The LOD and LOQ for each analyte under the present chromatographic conditions were determined at a signal-to-noise ratio (S/N) of about 3 and 10, respectively.

### Precision, repeatability, accuracy, and stability

Precision of the HPLC-ELSD method was determined by evaluating the repeatability of intra-day and inter-day measurements at low, middle and high level. Three known concentration solutions of 13 standards were prepared. For intra-day variability test, the mixed standards solutions were analyzed for six replicates within 1 day, while for inter-day variability test, the solutions were examined in duplicates for three successive days. Variations were expressed by the relative standard deviations (RSD) for intra- and inter-day. Repeatability of the developed method was confirmed with preparation and analysis of six parallel samples. In order to check the accuracy of the developed method, recovery of the investigated components was carried out. Known amounts of the mixed standards were added into the samples (0.25 g) and then the mixture was extracted, processed, and analyzed in accordance with methods mentioned above. Three replicates were performed for the test. Average recoveries were determined by the equation recovery (percent) = 100 × (observed amount − original amount)/spiked amount, and relative standard deviation (RSD, percent) = 100 × standard deviation (SD)/mean. Stability was tested and analyzed at 0, 2, 4, 8, 16, 24, and 48 h.

### Data analysis

HCA, PCA and FA were performed by SPSS 16.0 for windows (SPSS Inc., Chicago IL, USA), which comprises a number of procedures-graphical, statistical, reporting, processing and tabulating procedures that can be used for fast data analysis. A method named as Ward’s method was applied and Squared Euclidean distance, a pattern similarity measure, was finally selected as measurement for HCA.

## Results

### Optimization of sample preparation

**Fig. 2:**
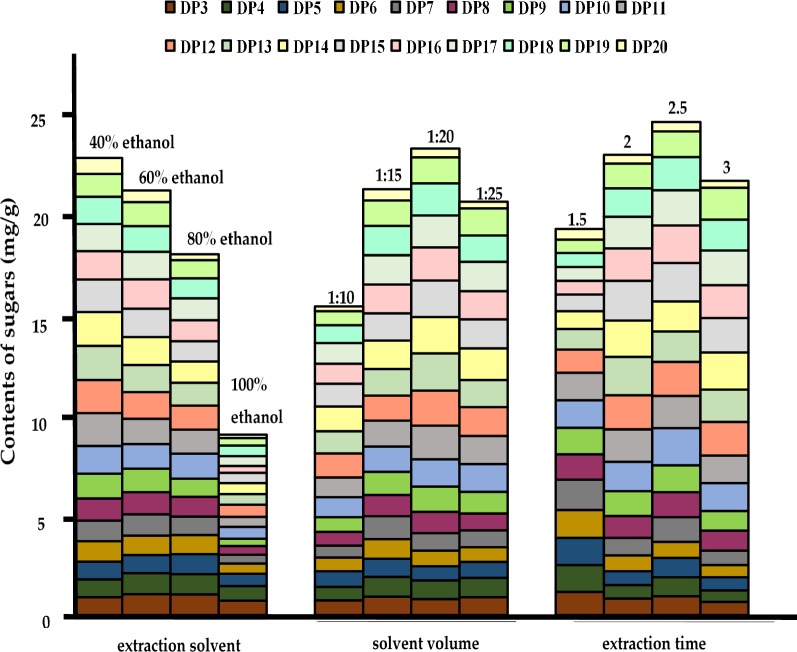
Effects of extraction solvent, solvent volume and extraction time on the extraction efficiency of FOS with different degrees of polymerization (DP) in *A. lancea* (A9)

The extraction solvents, extraction solvent volume, extraction time, which directly influence the extraction yields of FOS, were optimized. Refluxing extraction procedure was optimized using sample *A. lancea* (A9). The amount of 18 investigated compounds (DP 3-20) was used as the marker for evaluation of extraction efficiency. Different extraction solvents (40, 60, 80, and 100% ethanol) were investigated using univariate approach. Figure 2 showed that the extraction efficiency of FOS by 40, and 60% ethanol were obviously higher than that of 80, and 100% ethanol. In order to reduce the extraction of water-soluble impurities such as polysaccharides and other macromolecules, the 60% ethanol was adopted for sample preparation [18, 23]. Different ratio of solid to liquid, 1:10, 1:15, 1:20 and 1:25, were compared for the extraction of FOS in *A. lancea*. The results suggested that the extraction efficiency of FOS was obviously higher when solvent volume was 20-fold to sample material (v/w). We also have investigated the effect of extraction time (1.5, 2, 2.5, and 3 h). It turned out that the optimal extraction time of FOS was 2.5 h, whereas longer extraction time could not increase extraction yields of FOS. Therefore, the optimized refluxing extraction method was solvent, 60% ethanol; solvent volume, 20-fold to sample material (v/w); extraction time, 2.5 h.

### Method validation

The method of HPLC-ELSD was validated for the quantification. Linearity, regression, and linear ranges of the 13 analytes were determined by the developed HPLC method. The calibration curves with the R^2^, linear range and regression equation, LOD and LOQ of 13 targeted analytes are listed in Table 2. The results indicated the calibration curves of the 13 analytes had good linearity (R^2^ > 0.9993) between the analytes concentrations and their peak areas within the test ranges, and their LODs and LOQs were in the ranges of 0.01–0.04 mg/mL and 0.04–0.14 mg/mL, respectively (Table 2). Moreover, for intra-day precision at low, middle and high concentration, RSDs of the peak area were less than 4.43% and RSDs of the retention time were less than 0.50%; for inter-day precision, the corresponding RSDs were less than 10.91% and 0.48% (Table 3). The recoveries of 13 analytes were between 83.16 and 99.66%, and the analytes were stable in 60% ethanol solution during the tested period (Table 4). Repeatability with RSD less than 2.66% suggested that the developed method had a good repeatability for the quantitative evaluation of the analytes. These results showed that the developed HPLC-ELSD method was sensitive, precise and accurate for quantitative analysis of 13 inulin-type FOS (DP 3-15) in Atractylodis rhizome.

**Table 2:** Linear regression data and limits of detection and quantitation for FOS DP 3-15

Analyte	Regression equation	R^2^	Linear range (mg/mL)	LOD (mg/mL)	LOQ (mg/mL)
DP3	y = 1.8145x + 1.1883	0.9993	0.37–2.99	0.04	0.14
DP4	y = 1.7386x + 1.7843	0.9999	0.09–2.63	0.02	0.07
DP5	y = 1.7326x + 1.8361	0.9999	0.10–2.64	0.02	0.08
DP6	y = 1.7330x + 1.7222	0.9997	0.09–2.63	0.02	0.08
DP7	y = 1.7224x + 1.9625	0.9999	0.09–2.62	0.01	0.05
DP8	y = 1.7239x + 1.9418	0.9999	0.09–2.63	0.01	0.04
DP9	y = 1.7325x + 1.8667	0.9999	0.10–2.64	0.02	0.07
DP10	y = 1.7297x + 1.8748	0.9997	0.10–2.65	0.02	0.07
DP11	y = 1.7616x + 1.9440	0.9997	0.10–2.66	0.01	0.04
DP12	y = 1.8066x + 1.9046	0.9996	0.10–2.30	0.01	0.05
DP13	y = 1.8212x + 1.8248	0.9997	0.10–2.64	0.02	0.07
DP14	y = 1.8534x + 1.6928	0.9993	0.09–2.62	0.02	0.08
DP15	y = 1.9168x + 1.3978	0.9997	0.19–2.60	0.04	0.13

**Table 3:** Precision for FOS DP 3-15

Analyte	1.35 mg/mL				1.65 mg/mL				1.95 mg/mL			
	Peak area (% RSD)		Retention time (% RSD)		Peak area (% RSD)		Retention time (% RSD)		Peak area (% RSD)		Retention time (% RSD)	
	Intraday (n = 6)	Interday (n = 6)	Intraday (n = 6)	Interday (n = 6)	Intraday (n = 6)	Interday (n = 6)	Intraday (n = 6)	Interday (n = 6)	Intraday (n = 6)	Interday (n = 6)	Intraday (n = 6)	Interday (n = 6)
DP3	1.00	8.77	0.50	0.30	0.29	8.18	0.28	0.11	2.45	7.72	0.16	0.18
DP4	1.21	7.03	0.49	0.44	0.35	7.61	0.27	0.44	1.88	10.03	0.16	0.46
DP5	0.46	9.51	0.47	0.48	0.40	8.16	0.27	0.12	2.13	10.36	0.15	0.21
DP6	1.11	7.18	0.47	0.31	0.86	8.68	0.26	0.10	2.27	10.84	0.14	0.20
DP7	0.90	8.62	0.45	0.32	0.93	8.46	0.26	0.11	2.22	10.57	0.13	0.18
DP8	1.02	7.97	0.44	0.32	0.71	7.31	0.25	0.11	2.29	9.96	0.12	0.16
DP9	0.76	6.93	0.42	0.33	0.57	7.51	0.24	0.11	2.67	10.02	0.11	0.14
DP10	0.87	8.62	0.42	0.32	0.56	6.39	0.23	0.10	2.67	10.11	0.10	0.12
DP11	4.29	7.41	0.39	0.34	2.10	7.16	0.20	0.11	2.96	9.79	0.11	0.11
DP12	3.84	7.25	0.41	0.32	0.41	7.30	0.22	0.11	2.80	9.82	0.09	0.10
DP13	1.39	9.85	0.39	0.32	0.93	7.98	0.20	0.10	3.86	10.28	0.08	0.09
DP14	1.24	9.41	0.38	0.32	1.01	9.37	0.20	0.08	3.23	10.91	0.07	0.08
DP15	2.96	6.92	0.37	0.31	2.80	7.29	0.20	0.10	4.43	5.74	0.07	0.08

**Table 4:** Repeatability, Stability, and Accuracy for FOS DP 3-15

Analyte	Repeatability (n = 6)		Stability (48 h)	Accuracy (n = 3)	
	Content (mg/g)	RSD (%)	RSD (%)	Recovery (%)	RSD (%)
DP3	6.13	1.07	3.27	90.31	2.29
DP4	2.17	2.66	2.63	83.74	1.43
DP5	2.15	2.07	4.93	84.75	0.70
DP6	3.32	1.66	4.97	83.16	2.54
DP7	2.56	1.43	3.82	91.47	3.57
DP8	3.02	2.04	4.89	84.79	0.89
DP9	3.88	2.27	3.45	96.48	3.38
DP10	4.81	1.72	4.98	94.77	0.87
DP11	4.91	1.45	3.80	90.71	3.66
DP12	5.57	2.26	4.52	90.97	5.10
DP13	6.65	1.81	4.80	93.72	5.98
DP14	7.88	2.21	4.69	91.86	5.48
DP15	10.46	1.68	4.84	99.66	5.26

### Quantitative determination of FOS in plants

**Fig. 3:**
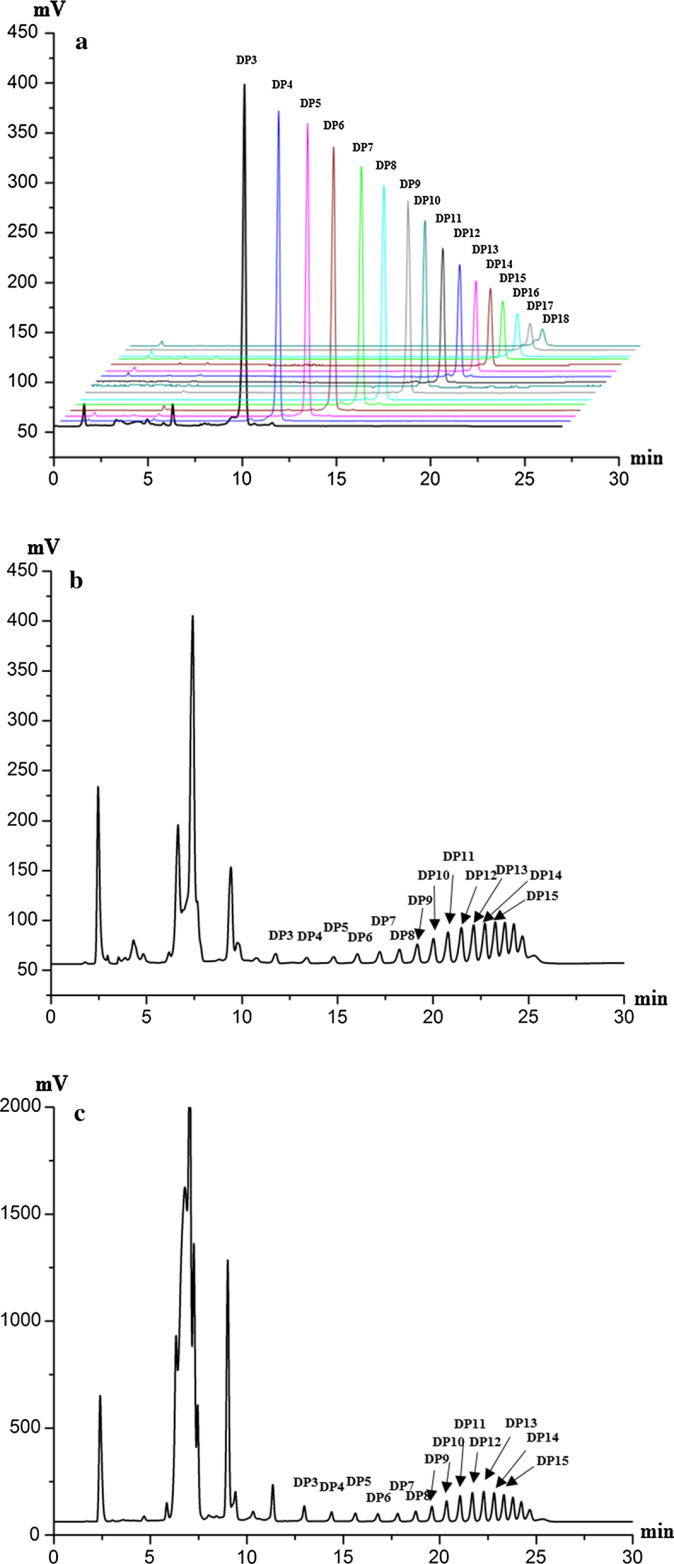
HPLC-ELSD chromatograms of **a** mixed standards and samples of **b** *A. lancea* and **c** *A. chinensis*

**Fig. 4:**
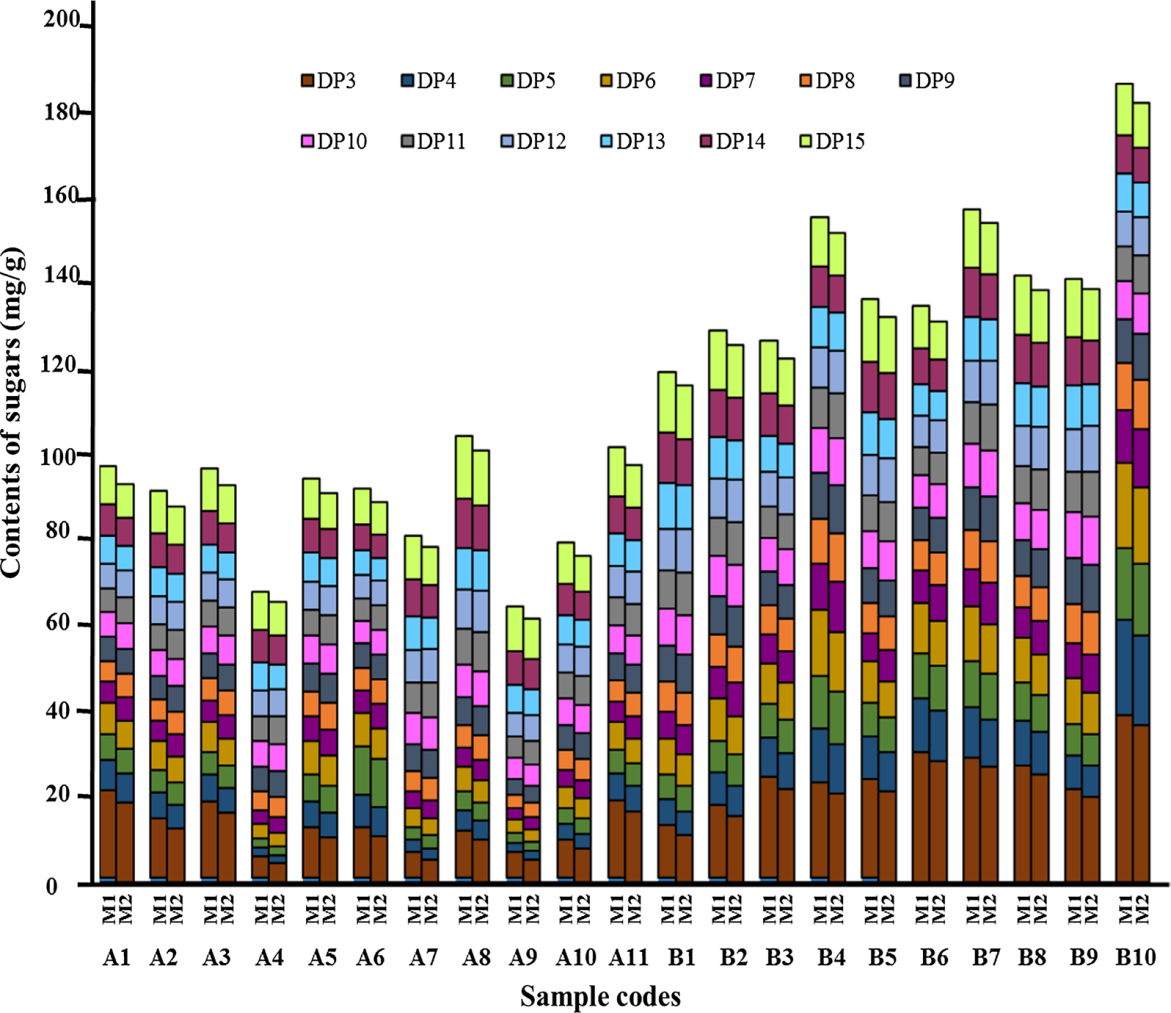
Comparison for the content (mg/g) of FOS with different degree of polymerization (DP) in *A. lancea* and *A. chinensis* calculated based on individual calibration curve (Method 1) and calculated based on the calibration curve of DP 5 (Method 2). The sample codes are the same as listed in Table 1

The established HPLC-ELSD method was applied for analysis of inulin-type FOS in different samples of *A. lancea* and *A. chinensis*. Typical HPLC-ELSD chromatograms of mixed standards and crude extracts were shown in Fig. 3. The identification of the investigated compounds was carried out by comparison of their retention time with those obtained by injecting standards under the same conditions. Besides the DP 3-15, FOS with higher DP were also found in *A. lancea* and *A. chinensis* (Fig. 3b, c). DP 16-18 were identified by chemical standards separated in our lab, which the amount was limited and therefore did not validate for the quantification method. DP 3-15 were validated for the quantification analysis. The 13 investigated oligosaccharides in *A. lancea* and *A. chinensis* were well separated using the developed HPLC method. The content of these 13 investigated oligosaccharides were calculated based on individual calibration curve (Method 1), and the content were summarized in Table 5. As shown in Table 5 and Fig. 4, the content of FOS in *A. chinensis* were relatively higher than that of *A. lancea*. The content of the 13 analytes differed among these two species, which might lead to variances in the pharmacologic actions, even their therapeutic effects. Thus, determination of multiple components is essential for the quality evaluation of Atractylodis rhizome for both natural and cultured products.

**Table 5:** Comparison for the content (mg/g) of FOS with different degree of polymerization (DP) in *A. lancea* and *A. chinensis* calculated based on individual calibration curve (Method 1, M1) and calculated based on the calibration curve of DP 5 (Method 2, M2)

Sample	Content (mg/g)																				
	DP3			DP4			DP5			DP6			DP7			DP8			DP9		
	M1	M2	PD (%)	M1	M2	PD (%)	M1	M2	PD (%)	M1	M2	PD (%)	M1	M2	PD (%)	M1	M2	PD (%)	M1	M2	PD (%)
A1	20.42	18.68	8.91	7.15	6.65	7.24	5.97	5.97	–^a^	7.31	6.54	11.16	4.92	5.42	9.64	4.94	5.36	8.10	5.64	5.87	4.07
A2	13.97	12.55	10.70	5.89	5.48	7.30	5.37	5.37	–	6.74	6.02	11.16	4.69	5.17	9.67	4.93	5.35	8.10	5.71	5.94	4.07
A3	18.01	16.37	9.50	6.11	5.68	7.29	5.31	5.31	–	7.00	6.26	11.16	5.01	5.51	9.63	5.18	5.62	8.08	5.93	6.18	4.07
A4	5.13	4.59	11.05	1.96	1.82	7.68	2.05	2.05	–	3.46	3.09	11.18	3.27	3.61	9.88	4.36	4.73	8.16	5.74	5.97	4.07
A5	11.80	10.51	11.49	6.14	5.71	7.29	6.25	6.25	–	7.86	7.03	11.16	5.69	6.26	9.56	5.80	6.28	8.02	6.47	6.74	4.07
A6	11.99	10.70	11.42	7.36	6.85	7.23	11.23	11.23	–	7.86	7.04	11.01	5.41	5.93	9.17	5.29	5.68	7.11	5.52	5.76	4.26
A7	6.11	5.39	12.58	2.79	2.58	7.56	3.07	3.07	–	4.46	3.98	11.17	3.73	4.12	9.80	4.72	5.12	8.12	6.45	6.72	4.07
A8	11.20	9.96	11.74	4.71	4.37	7.38	4.34	4.34	–	5.79	5.18	11.17	4.39	4.84	9.71	5.17	5.61	8.08	6.62	6.90	4.07
A9	6.13	5.35	13.58	2.17	1.98	9.15	2.15	2.15	–	3.32	2.98	10.79	2.56	2.81	9.31	3.02	3.25	7.33	3.88	4.04	4.04
A10	9.01	7.93	12.76	3.72	3.46	7.46	3.68	3.68	–	4.99	4.47	11.17	3.92	4.32	9.78	4.58	4.97	8.14	5.87	6.12	4.07
A11	18.15	16.51	9.46	6.38	5.94	7.28	5.37	5.37	–	6.49	5.81	11.16	4.70	5.17	9.67	5.13	5.56	8.08	6.12	6.38	4.07
B1	12.32	11.00	11.29	5.96	5.54	7.30	5.91	5.91	–	8.36	7.48	11.16	6.25	6.87	9.50	6.96	7.54	7.93	8.45	8.81	4.07
B2	17.00	15.42	9.77	7.72	7.18	7.21	7.40	7.40	–	9.78	8.75	11.16	7.27	7.99	9.41	7.71	8.34	7.88	8.96	9.34	4.07
B3	23.72	21.85	8.20	8.99	8.37	7.16	7.89	7.89	–	9.55	8.54	11.16	6.70	7.36	9.46	6.93	7.51	7.93	7.66	7.98	4.07
B4	22.43	20.61	8.46	12.38	11.54	7.05	12.29	12.29	–	15.63	13.98	11.14	10.75	11.78	9.18	10.31	11.14	7.73	10.81	11.26	4.07
B5	23.14	21.29	8.32	9.81	9.14	7.13	8.08	8.08	–	9.50	8.50	11.16	6.59	7.25	9.47	7.15	7.74	7.91	8.09	8.43	4.07
B6	30.35	28.28	7.04	12.65	11.79	7.04	10.50	10.50	–	11.69	10.45	11.15	7.56	8.31	9.39	7.12	7.71	7.92	7.63	7.94	4.07
B7	28.96	26.93	7.26	11.87	11.06	7.06	10.70	10.70	–	12.84	11.48	11.15	8.82	9.67	9.30	9.02	9.75	7.80	10.08	10.50	4.07
B8	27.22	25.24	7.55	10.49	9.77	7.10	8.81	8.81	–	10.59	9.47	11.15	7.10	7.80	9.43	7.23	7.82	7.91	8.43	8.78	4.07
B9	21.81	20.01	8.60	7.91	7.36	7.20	7.27	7.27	–	10.68	9.55	11.15	8.17	8.97	9.34	9.20	9.94	7.79	10.65	11.09	4.07
B10	38.97	36.75	5.86	22.38	20.90	6.84	16.80	16.80	–	19.70	17.62	11.14	12.46	13.65	9.10	10.81	11.68	7.71	10.21	10.63	4.07
Cos(θ)		0.99986			1.00000			–			1.00000			1.00000			1.00000			1.00000	

Sample	Content (mg/g)																				
	DP10			DP11			DP12			DP13			DP14			DP15			Total		
	M1	M2	PD (%)	M1	M2	PD (%)	M1	M2	PD (%)	M1	M2	PD (%)	M1	M2	PD (%)	M1	M2	PD(%)	M1	M2	PD (%)
A1	5.66	5.97	5.35	5.60	6.17	9.55	5.86	6.09	3.86	6.36	5.90	7.36	7.29	6.42	12.77	9.02	7.98	12.26	96.14	93.02	3.30
A2	6.01	6.34	5.34	6.03	6.64	9.67	6.43	6.71	4.26	6.80	6.34	7.02	7.91	7.00	12.21	9.98	8.78	12.83	90.46	87.69	3.11
A3	6.24	6.58	5.34	6.04	6.65	9.67	6.38	6.66	4.22	6.64	6.19	7.13	7.78	6.88	12.32	9.97	8.77	12.80	95.6	92.66	3.12
A4	6.00	6.33	5.34	5.89	6.49	9.63	6.03	6.27	3.98	6.42	5.97	7.31	7.50	6.61	12.57	9.10	7.98	13.16	66.91	65.51	2.11
A5	6.50	6.85	5.21	6.09	6.71	9.68	6.53	6.82	4.32	6.97	6.51	6.89	7.65	6.76	12.43	9.63	8.43	13.29	93.38	90.86	2.74
A6	5.42	5.71	5.44	5.22	5.72	9.14	5.45	5.75	5.36	5.66	5.41	4.52	6.13	5.49	11.02	8.36	7.51	10.71	90.9	88.78	2.36
A7	7.26	7.66	5.31	7.11	7.86	9.94	7.54	7.92	4.93	7.83	7.35	6.29	8.59	7.65	11.63	10.16	8.93	12.72	79.82	78.35	1.86
A8	7.62	8.03	5.30	8.24	9.12	10.19	9.16	9.71	5.77	9.87	9.37	5.11	11.50	10.45	9.61	14.49	12.96	11.16	103.1	100.84	2.22
A9	4.81	5.08	5.46	4.91	5.43	10.05	5.57	5.89	5.58	6.65	6.09	8.79	7.88	7.19	9.16	10.46	9.31	11.63	63.51	61.55	3.13
A10	6.22	6.56	5.34	6.08	6.69	9.68	6.43	6.71	4.26	6.77	6.31	7.04	7.50	6.62	12.57	9.45	8.36	12.27	78.22	76.20	2.62
A11	6.60	6.96	5.33	6.59	7.27	9.81	7.23	7.58	4.75	7.72	7.24	6.37	8.69	7.75	11.55	11.34	9.88	13.76	100.51	97.42	3.12
B1	8.69	9.16	5.28	9.02	10.01	10.34	9.63	10.22	5.98	10.61	10.11	4.74	11.90	10.84	9.37	14.08	12.56	11.47	118.14	116.05	1.78
B2	9.30	9.80	5.27	8.85	9.81	10.31	9.31	9.87	5.84	9.71	9.22	5.19	11.02	9.98	9.90	13.73	12.21	11.73	127.76	125.31	1.94
B3	7.92	8.35	5.30	7.38	8.15	10.00	8.11	8.55	5.25	8.44	7.95	5.91	9.87	8.87	10.67	12.36	10.87	12.85	125.52	122.24	2.65
B4	10.56	11.13	5.25	9.40	10.43	10.41	9.39	9.96	5.87	9.31	8.82	5.41	9.59	8.60	10.87	11.41	9.94	13.70	154.26	151.48	1.82
B5	8.64	9.11	5.28	8.44	9.35	10.23	9.42	9.99	5.89	9.76	9.27	5.17	11.76	10.70	9.45	14.68	13.15	11.02	135.06	132.00	2.29
B6	7.48	7.88	5.31	6.71	7.41	9.85	7.19	7.54	4.73	7.41	6.94	6.58	8.29	7.36	11.88	9.93	8.74	12.72	134.51	130.85	2.76
B7	10.10	10.64	5.26	9.69	10.76	10.46	9.62	10.21	5.97	10.34	9.84	4.88	11.32	10.27	9.71	13.74	12.22	11.73	157.1	154.03	1.97
B8	8.65	9.12	5.28	8.69	9.63	10.28	9.32	9.88	5.84	9.92	9.43	5.09	11.29	10.24	9.74	13.87	12.35	11.62	141.61	138.34	2.34
B9	10.63	11.20	5.25	9.52	10.57	10.43	9.92	10.54	6.11	10.15	9.66	4.97	11.26	10.21	9.75	13.67	12.15	11.78	140.84	138.52	1.66
B10	8.98	9.47	5.28	8.04	8.90	10.15	8.32	8.78	5.36	8.74	8.25	5.74	9.04	8.07	11.28	11.91	10.43	13.24	186.36	181.93	2.41
Cos(θ)		1.00000			1.00000			0.99998			0.99995			0.99993			0.99996			0.99999	

^a^Not applicable

### Chemical characteristics of *A. lancea* and *A. chinensis*

**Fig. 5:**
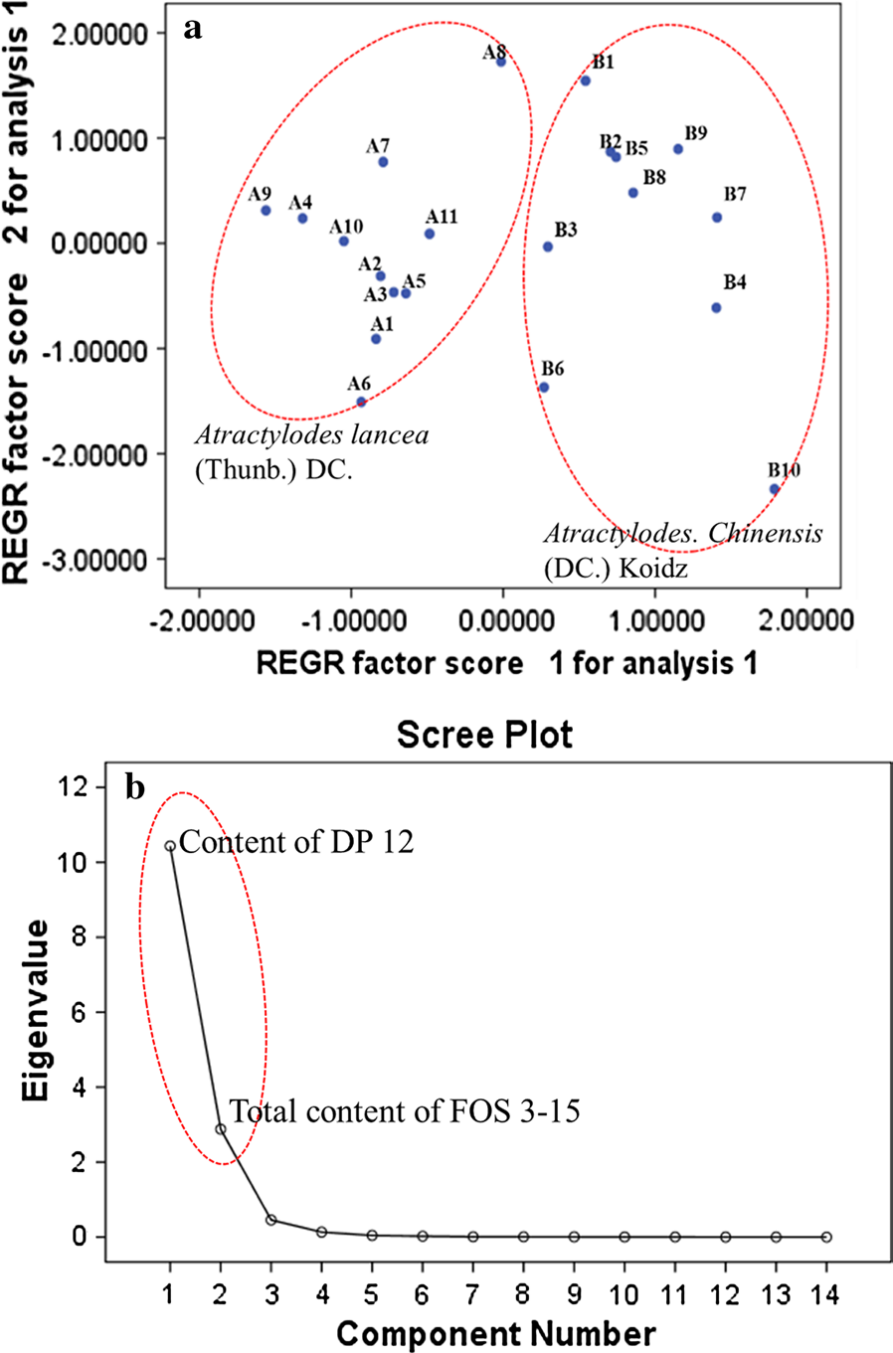
Scatter plot of PCA (**a**) and FA (**b**) of FOS with different degree of polymerization (DP) for *A. lancea* and *A. chinensis*. The sample codes are the same as listed in Table 1

In order to evaluate the differences of FOS in *A. lancea* and *A. chinensis* and identify the distinctive ingredients, PCA was performed. The PCA was carried out by using the content of 13 analytes derived from quantification analysis. The scatter points (Fig. 5a) showed that *A. lancea* and *A. chinensis* were well separated. The results indicated that significant difference existed in *A. lancea* and *A. chinensis*, which probably induce the different pharmacological effects.

FA was employed to identify the distinctive ingredients and reduce the markers for quality control of *A. lancea* and *A. chinensis*. Eventually, 2 markers including content of DP 12 and total content of FOS 3-15 were identified as the main distinctive elements, which were available for distinguishing and quality control of *A. lancea* and *A. chinensis* (Fig. 5b).

**Fig. 6:**
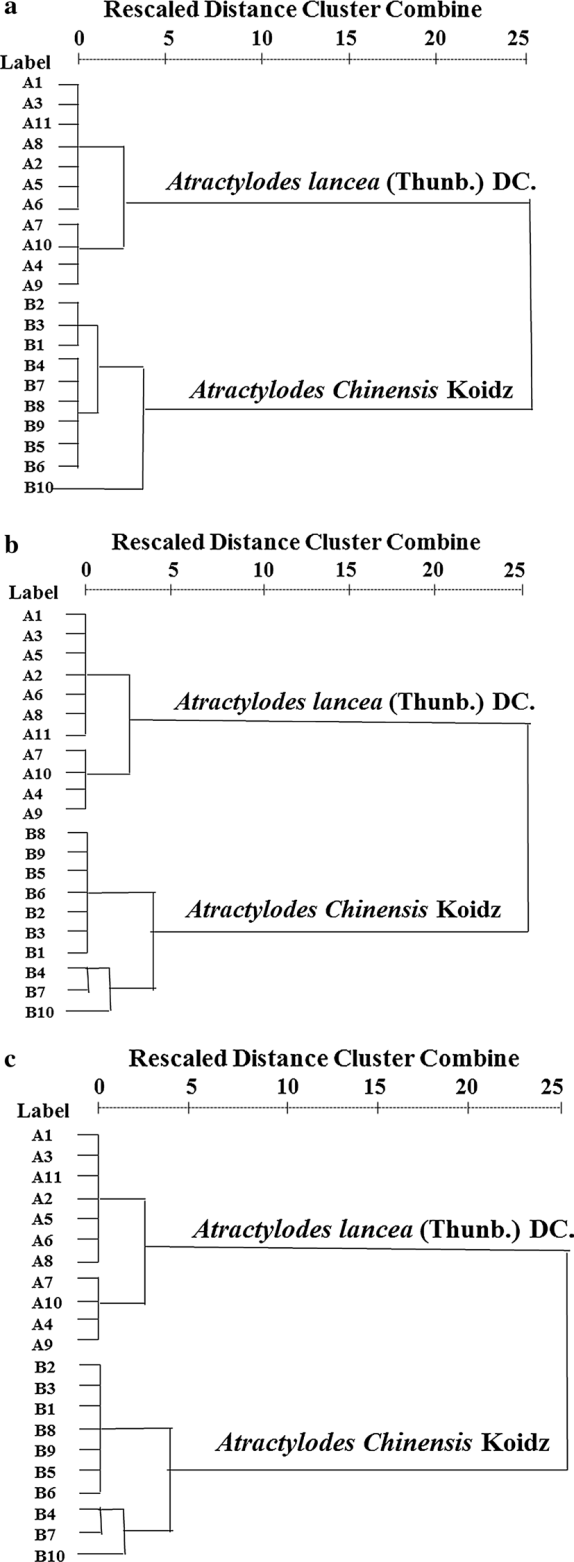
Dendrograms of hierarchical cluster analysis for 21 tested samples of *A. lancea* and *A. chinensis* based on **a** 13 investigated FOS calculated by individual calibration curve (Method 1), **b** 2 markers (content of DP 12, and total content of FOS 3-15) derived from their FA and **c** 13 investigated FOS calculated by calibration curves of DP 5 (Method 2). The sample codes were the same as in Table 1

Hierarchical cluster analysis of 21 selected samples of Atractylodis rhizome was performed using content of DP 3-15 (Fig. 6a) and 2 selected markers based on FA (Fig. 6b), respectively. Their results were very similar, *A. lancea* and *A. chinensis* were well separated, which were also in accordance with that of PCA. Therefore, the content of DP 12 and total content of FOS 3-15 could be used as markers for quality control of Atractylodis rhizome. Actually, the separation of high DP FOS such as DP 12 is difficult and time-consuming, therefore an alternative method for accurate determination of total content of FOS 3-15 is an ideal means to solve the shortage of reference compound.

### Quantification method assessment

Take the advantage of similar structure of FOS and homogeneous response on ELSD [24, 25], we have calculated the FOS 3-15 based on the calibration curve of DP 3, 4 and 5, respectively (Method 2). In order to evaluate the method feasibility, percent difference (PD), and cosine similarity between two vectors Cos(θ), were employed [26, 27]. The calculation of PD is as follows:

1 $$ 100\, \times \,{{\left( {{\mid }x1\, - \,x2{\mid }} \right)} \mathord{\left/ {\vphantom {{\left( {{\mid }x1\, - \,x2{\mid }} \right)} {\left[ {{{\left( {x1\, + \,x2} \right)} \mathord{\left/ {\vphantom {{\left( {x1\, + \,x2}\right)}2}} \right. \kern-0pt} 2}} \right]}}} \right. \kern-0pt} {\left[ {{{\left( {x1\, + \,x2} \right)} \mathord{\left/ {\vphantom {{\left( {x1\, + \,x2} \right)}2}} \right. \kern-0pt} 2}} \right]}} $$

where x1 and x2 are the content produced by Methods 1 and 2.

The calculation of Cos(θ) is as the following equation:

2 $$ { \cos }\left(\uptheta \right)\left( {{\text{X}}, {\text{Y}}} \right)\, = \,\frac{{\mathop \sum \nolimits_{i = 1}^{n} XiYi}}{{\sqrt {\mathop \sum \nolimits_{i = 1}^{n} \left( {Xi} \right)^{2} } \, \times \,\sqrt {\mathop \sum \nolimits_{i = 1}^{n} \left( {Yi} \right)^{2} } }} $$

where X and Y are the content produced by Methods 1 and 2, and n is the number of data sets [26, 27].

Based on the calibration curve of DP 3 and 4, the average PDs of FOS 3-15 were up to 26.00% and 25.84%, then the corresponding Cos(θ) were as low as 0.99797 and 0.99785, respectively. Therefore, we calculated the FOS 3-15 based on the calibration curve of DP 5. The results showed that the average PDs of 13 analytes were all less than 13.76% and the Cos(θ) were 0.99986, 1.00000, 1.00000, 1.00000, 1.00000, 1.00000, 1.00000, 1.00000, 0.99998, 0.99995, 0.99993, 0.99996 and 0.99999 (Table 5), which demonstrated that the similarities of pairwise arrays between Methods 1 and 2 were very high. In addition, the HCA analysis based on the total content of FOS 3-15 calculated by DP 5-based quantification (Fig. 6c) also showed high degree of consistency with the results based on individual calibration curve (Fig. 6a). The results indicated that DP 5-based quantitative method could be adopted to simplify the analytical procedure for quantification of all the investigated components.

Actually, the variation of analytes’ content among different samples derived from natural material was usually high, which significantly indicated that the quality control of FOS in Atractylodis rhizome was crucial. Also, the variation of content was much higher than the quantification errors using DP 5 for calculation and the quantification errors should be acceptable for quality control of Atractylodis rhizome.

## Conclusions

In this study, a HPLC-ELSD quantification method based on the individual calibration curve (Method 1) has been developed. Coupled with chemometrics analysis, we demonstrated that 2 markers including content of DP12 and total content of DP 3-15 could be used as the main distinctive elements, which were available for distinguishing and quality control of Atractylodis rhizome. Actually, the separation and purification of high DP FOS, such as DP 12, is still a challenge because of high polarity. However, the quantitative evaluation of total content of DP 3-15 based on low DP standards is relatively easy to implement. DP 5 could be purified on Bio-Gel P-2 column eluted with water at the flow rate of 0.3 mL/min according to our previous work and at present DP 5 has been commercialized. Thus, DP 5-based qualification of multiple analytes (Method 2) is especially suitable for the quality control of Atractylodis rhizome. In addition, using relative content of 13 oligosaccharides based on Method 2 as input data matrix, *A. lancea* and *A. chinensis* were well separated, which were also in accordance with that of HCA based on Method 1. Therefore, DP 5-based qualification and quantitative evaluation of FOS in Atractylodis rhizome was demonstrated to be credible for solving the shortage of reference compounds and improving the quality control of FOS in Atractylodis rhizome as well as other herbs containing FOS.

## Data Availability

All data and materials are all provided.

## Abbreviations

*A. lancea*: *Atractylodes lancea* (Thunb.) DC
*A. chinensis*: *Atractylodes chinensis* (DC.) Koidz
FOS: fructooligosaccharides
DP: degree of polymerization

## References

[1] Wu KC, Kao C-P, Ho Y-L (2017). Quality control of the root and rhizome of *Helminthostachys zeylanica* (Daodi-Ugon) by HPLC using quercetin and ugonins as markers. Molecules.

[2] Kuo X, Jiang JS, Feng ZM, Yang YN (2016). Bioactive sesquiterpenoid and polyacetylene glycosides from *Atractylodes lancea*. J Nat Prod.

[3] Ouyang Z, Zhang L, Zhao M (2012). Identification and quantification of sesquiterpenes and polyacetylenes in *Atractylodes lancea* from various geographical origins using GC–MS analysis. Rev Bras Farmacogn Braz J Pharmacogn.

[4] Yan Y, Jia TZ, Cai Q, Jiang N (2015). Comparison of the anti-ulcer activity between the crude and bran-processed Atractylodes lancea in the rat model of gastric ulcer induced by acetic acid. J Ethnopharmacol.

[5] Guo FQ, Huang LF, Zhou SY, Zhang TM, Liang YZ (2006). Comparison of the volatile compounds of Atractylodes medicinal plants by headspace solid-phase microextraction-gas chromatography–mass spectrometry. Anal Chim Acta.

[6] Liu Q, Zhang S, Yang X, Wang R (2016). Differentiation of essential oils in *Atractylodes lancea* and *Atractylodes koreana* by gas chromatography with mass spectrometry. J Sep Sci.

[7] Kuo X, Yang YN, Feng ZM, Jiang JS (2016). Six new compounds from *Atractylodes lancea* and their hepatoprotective activities. Bioorg Med Chem Lett.

[8] Zhou JY, Yuan J, Li X, Ning YF (2016). Endophytic bacterium-triggered reactive oxygen species directly increase oxygenous sesquiterpenoid content and diversity in *Atractylodes lancea*. Appl Environ Microbiol.

[9] Liu J, Chen X, Yue C, Hou R, Chen J (2015). Effect of selenylation modification on immune-enhancing activity of *Atractylodes macrocephala* polysaccharide. Int J Biol Macromol.

[10] Wang J, Sporns P, Low NH (1999). Analysis of food oligosaccharides using MALDI–MS: quantification of fructooligosaccharides. J Agric Food Chem.

[11] Seipert RR, Barboza M (2008). Analysis and quantitation of fructooligosaccharides using matrix-assistedlaser desorption/ionization fourier transform ion cyclotron resonance mass spectrometry. Anal Chem.

[12] Reiffova K, Nemcova R (2006). Thin-layer chromatography analysis of fructooligosaccharides in biological samples. J Chromatogr A.

[13] Karboune S, Appanah N, Khodaei N, Tian F (2018). Enzymatic synthesis of fructooligosaccharides from sucrose by endo-inulinase-catalyzed transfructosylation reaction in biphasic systems. Process Biochem.

[14] Fermin CC, Borja O, Carlos JA, Andrea A, Maria DS (2016). Fructooligosaccharides exert intestinal anti-inflammatory activity in the CD4+ CD62L+ T cell transfer model of colitis in C57BL/6J mice. Eur J Nutr.

[15] Adeela Y, Sadiq BM, Muhammad Y (2015). Compositional analysis of developed whey based fructooligosaccharides supplemented low- calorie drink. J Food Sci Technol.

[16] Tian T, Freeman S, Mark Corey J, German B (2017). Chemical characterization of potentially prebiotic oligosaccharides in brewed coffee and spent coffee grounds. J Agric Food Chem.

[17] Tobias P, Christoph B, Hartwig S, Melanie S (2017). Comparison of high performance anion exchange chromatography with pulsed amperometric detection (HPAEC-PAD) and ultra-high performance liquid chromatography with evaporative light scattering (UHPLC-ELSD) for the analyses of fructooligosaccharides in onion (*Allium cepa* L.). J Food Compost Anal.

[18] Benkeblia N (2013). Fructooligosaccharides and fructans analysis in plants and food crops. J Chromatogr A.

[19] Li J, Cheong KL, Zhao J, Hu DJ, Chen XQ (2013). Preparation of inulin-type fructooligosaccharides using fast protein liquid chromatography coupled with refractive index detection. J Chromatogr A.

[20] Paredes LR, Smiderle FR, Santana-Filho AP (2018). Yacon fructans (*Smallanthus sonchifolius*) extraction, characterization and activation of macrophages to phagocyte yeast cells. Int J Biol Macromol.

[21] Lopes SMS, Francisco MG, Higashi B (2016). Chemical characterization and prebiotic activity of fructo-oligosaccharides from *Stevia rebaudiana* (Bertoni) roots and in vitro adventitious root cultures. Carbohydr Polym.

[22] Prasanna Kumar V, Harish Prashanth KV (2015). Structural analyses and immunomodulatory properties of fructo-oligosaccharides from onion (*Allium cepa*). Carbohydr Polym.

[23] Li J, Liu X, Zhou B, Zhao J (2013). Determination of Fructooligosaccharides in Burdock Using HPLC and microwave-assisted extraction. J Agric Food Chem.

[24] Arndt JH, Macko T, Bruell R (2013). Application of the evaporative light scattering detector to analytical problems in polymer science. J Chromatogr A.

[25] Mojsiewicz-Pienkowska K (2009). On the issue of characteristic evaporative light scattering detector response. Crit Rev Anal Chem.

[26] Li SP, Qiao CF, Chen YW, Zhao J (2013). A novel strategy with standardized reference extract qualification and single compound quantitative evaluation for quality control of *Panax notoginseng* used as a functional food. J Chromatogr A.

[27] Lv G-P, De-Jun H, Zhou YQ, Zhang QW (2018). Preparation and application of standardized typical volatile components fraction from turmeric (*Curcuma longa* L.) by supercritical fluid extraction and step molecular distillation. Molecules.

